# Effects of Increasing Temperature on Bacterial Community Diversity in Mixed Stands of *Artemisia argyi* and *Solidago canadensis* in Eastern China

**DOI:** 10.3390/microorganisms12122415

**Published:** 2024-11-25

**Authors:** Haochen Yu, Guangqian Ren, Zhiyun Huang, Shanshan Qi, Biying Zhao, Xue Fan, Zhaoqi Zhu, Zhicong Dai, Daolin Du

**Affiliations:** 1 School of Emergency Management, School of Environment and Safety Engineering, Jiangsu University, Zhenjiang 212013, China; yhc960618@gmail.com (H.Y.);; 2 Jiangsu Province Engineering Research Center of Green Technology and Contingency Management for Emerging Pollutants, Jiangsu University, Zhenjiang 212013, China; 3 School of Agricultural Engineering, Jiangsu University, Zhenjiang 212013, China; 4 International Genome Centre, Jiangsu University, Zhenjiang 212013, China; 5 Institute of Environment and Ecology, School of the Environment and Safety Engineering, Jiangsu University, Zhenjiang 212013, China; 6 Jiangsu Collaborative Innovation Center of Technology and Material of Water Treatment, Suzhou University of Science and Technology, Suzhou 215009, China; 7 Jingjiang College, Jiangsu University, Zhenjiang 212013, China

**Keywords:** co-occurrence network, plant invasion community, random forest, soil bacteria

## Abstract

Global climate change and invasive plants significantly impact biodiversity and ecosystem functions. This study focuses on the effects of progressive warming on microbial communities within the *Solidago canadensis* invasion community, simulated through six stages of invasion progression, from minimal to dominant *S. canadensis* presence alongside native *Artemisia argyi*, in bulk soils collected from a natural habitat and cultivated under controlled greenhouse conditions. Utilizing high-throughput sequencing and microbial community analysis on 72 samples collected from the *S. canadensis* invasion community, the shifts in soil microbiota under varying warming scenarios were investigated (+0 °C, +1.15 °C and +1.86 °C). We observed significant shifts in invasion community soil bacteria in response to warming, with Acidobacteria, Actinobacteria, and others showing distinct responses between baseline and warmed conditions, while groups like Chlorobi and Cyanobacteria only differed significantly at higher temperature extremes. The random forests algorithm identified 14 taxa as biomarkers and a model was established to correlate *S. canadensis* invasion community soil microbiota with progressive warming. Co-occurrence network analysis revealed that moderate warming enhances microbial connectivity and the presence of a super-generalist, ASV 1160. However, further warming disrupts these networks by eliminating key generalists, revealing a potential reduction in network stability and diversity. These findings illuminate the dynamic responses of microbes in *S. canadensis* invasion community soil to varying temperature regimes, suggesting a model for successional dynamics and offering a deeper comprehension of microbial community shifts amid climatic fluctuations. This study delineates how warming significantly reshapes the soil microbial composition, potentially impacting *S. canadensis*’s invasion success unfavorably, thereby highlighting the importance of considering microbial dynamics in ecological management.

## 1. Introduction

Human activities, particularly fossil fuel combustion and land use changes, have led to significant global climate change, marked by a 0.78 °C increase in global surface temperature since industrialization. This temperature is projected to rise by 1.5–4.5 °C by century’s end [1]. Such changes, alongside globalization, exacerbate biological invasions [2,3,4,5]. Climate change primarily drives the spread of invasive alien plants (IAPs) [6,7,8], which disrupt biodiversity [9], the functioning of ecosystems [10], human health and well-being [11], and the economy [12].

Climate warming affects plant performance mainly by altering source–sink interactions and species’ thermal requirements [13,14]. This warming may allow exotic species from warmer climates to spread to areas where cold conditions previously limited their competitiveness against native species [15]. Additionally, warming influences soil microbial communities’ diversity, structure, and activity [16], which are integral to biogeochemical cycles. Understanding the impact of climate change on soil microbes is therefore globally significant [17]. Invasion mechanisms involve significant changes in soil microbes, enhancing invaders’ chances of establishing themselves in new environments [18,19,20]. Established theories of plant invasion [21] include the enemy release hypothesis (ERH) [22,23,24,25], accumulation of local pathogens (ALP) [26], enhanced mutualist hypothesis (EMH) [27], and plant–soil feedback [28,29]. All evidence unequivocally supports the soil microbiota as the main factor responsible for plant establishment and success.

**Figure 1:**
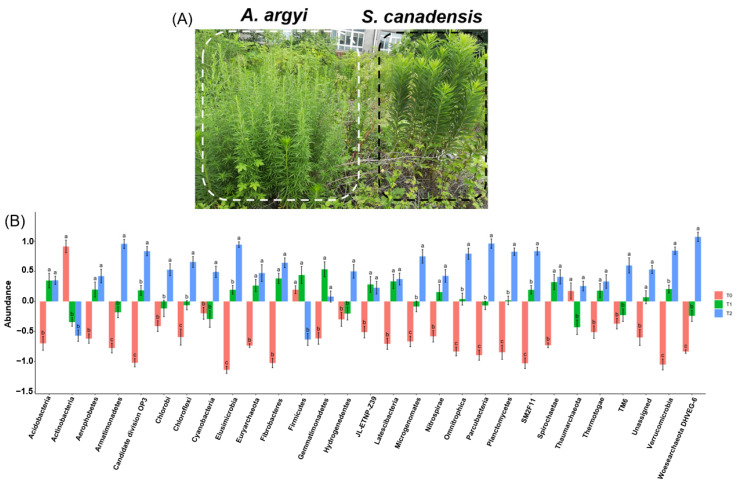
*S. canadensis* invasion community microbial composition responses to varying warming treatments. (**A**) Native *A. argyi* (**left**) and invasive *S. canadensis* (**right**) co-occurring in field. (**B**) Bar chart representing change in abundance of various microbial phyla across three temperature treatments (T0, T1, T2), with statistically significant differences denoted by letters.

*Solidago canadensis* L. (Asteraceae) (*S. canadensis*), a North American native perennial with durable rhizomes, ranks among the top invasive alien species worldwide, thriving in Europe, New Zealand, Australia, and parts of Asia. Introduced to Shanghai in 1935 as ornamental flora, it subsequently adapted to the wild [30]. Currently, it dominates numerous Chinese regions, earning a reputation as a highly destructive invasive pest [31]. Climate warming impacts *S. canadensis*’s lifecycle, notably delaying the onset of first inflorescence buds, flowering, seed-setting, and dieback, while promoting seed germination in native over invasive *S. canadensis. Artemisia argyi* (*A. argyi*) is a perennial herb or small shrub characterized by a strong, distinct aroma. It belongs to the Asteraceae family, and *A. argyi* is a prominent species primarily found in temperate regions of the northern hemisphere, including Asia, Europe, and North America [32]. Similar to *S. canadensis*, *A. argyi* is a perennial plant in the Asteraceae family and shares a comparable ecological niche in China, where it is indigenous [33]. The distribution ranges of both *S. canadensis* and *A. argyi* overlap significantly across large areas in China (Figure 1A) [34].

By focusing specifically on the impact of warming on the soil microbes in the *S. canadensis* invasion community, this research aims to deepen our understanding beyond our previous study. Our earlier research [13] investigated the combined effects of warming and nitrogen deposition on the performance and competitiveness of *S. canadensis* with the existence of *A. argyi*. We now hypothesize that warming alone significantly contributes to its invasive success. The objectives are as follows: 1. To dissect the specific impact of warming on the invasion community soil microbiota associated with *S. canadensis* in its invasion community. 2. To explore how temperature changes influence the composition and function of soil microbial communities in the context of the invasion community. We predict that warming significantly alters the microbial community associated with the *S. canadensis* invasion community, leading to shifts in diversity and increased abundance of specific microbes aiding invasion. Such microbial community changes, induced by warming, are expected to boost *S. canadensis*’s invasiveness. This research seeks to link the warming-induced shifts in soil microbial communities to the enhanced invasive success of *S. canadensis*. By analyzing these microbial changes, we aim to provide evidence on how warming impacts the soil microbial communities within the *S. canadensis* invasion community.

## 2. Materials and Methods

### 2.1. Experimental Setup

The experiment started on 14 June 2017, and ended on 6 September 2017. Seeds of *S. canadensis* (S) and native *Artemisia argyi* Levl. et Van (A) were collected during autumn 2016 from a plant community where these two species dominate, located in the suburbs of Zhenjiang City, China (119°51′ E, 32°20′ N). On 15 May 2017, the seeds were individually sown on the surface of natural soil in garden pots (24 cm in diameter, 18 cm in height), with the soil being taken from the same locations where the seeds were collected. Throughout germination, the plants were watered daily with tap water. When the seedlings reached approximately 1 cm in height, their number was adjusted in garden pots to match the intended conditions for the mixed culture experiment.

The experiment was conducted at Jiangsu University’s greenhouse in Zhenjiang, China. The city has a subtropical monsoon climate, with an average temperature of 15.9 °C, 1101.4 mm of rainfall, and 1996.8 h of sunlight annually [35]. Positioned at the confluence of the Yangtze River and the southeastern coast of China, it serves as a hotspot for *S. canadensis*. The study examined the invasive *S. canadensis* (S) and native *A. argyi* (A), both belonging to the Asteraceae family and sharing habitats in China [36]. Average temperatures during the study for temperature treatment 1 (T1) and temperature treatment 2 (T2) were 30.62 ± 0.36 °C and 31.33 ± 0.37 °C, surpassing the control’s (T0) 29.47 ± 0.38 °C by 1.15 °C and 1.86 °C, respectively, mirroring the global temperature rise expected by 2050 [37]. Based on the above-described treatment conditions, the plants were destructively harvested after 83 days of exposure to warming. Details on community establishment are provided in the Supplementary Materials.

### 2.2. DNA Extraction, PCR Amplification, and Sequencing

To prepare the bulk soil samples for analysis, the undecomposed litter layer on the soil surface was first removed. Then, a sterilized shovel was used to scrape away the top 5 cm of soil. Using a flame-sterilized shovel, 10 g of soil was sampled and placed into sterile plastic bags. Any plant or animal remains, stones, or other impurities were carefully removed from the sample. The soil was crushed to break up larger clumps and passed through a 2 mm sieve. The sieved sample was then divided and stored in 2 mL cryovials for further analysis.

Total DNA isolation was extracted with a DNA kit (Omega Bio-Tek, Norcross, GA, USA), following the manufacturer’s instructions. The assessment of DNA integrity and yield was conducted using agarose gel electrophoresis and a Qubit fluorometer from Thermo Fisher Scientific Inc., Carlsbad, CA, USA. Amplification of the DNA utilized primer sets 338F and 806R, targeting the V3-V4regions of the 16S rRNA gene. The PCR products had electrophoresis for analysis, and the target bands were purified using a gel extraction kit from Omega Bio-Tek, USA. Genepioneer Biotechnologies (Nanjing, China) sequenced the 16S rRNA gene amplicons using an Illumina Miseq PE300 platform (San Diego, CA, USA), adhering to the manufacturer’s protocol for high-throughput sequencing.

### 2.3. Sequence Processing and Amplicon Sequence Variant (ASV) Clustering and Filtering

Raw sequence data were dereplicated using Vsearch to identify unique sequences [38], setting a minimum unique size of 30 for noise reduction and computational efficiency. ASV denoising was performed using the unoise3 algorithm in Usearch [39], followed by reference-based chimera detection against the Silva database [40]. Sequences were annotated for species using Vsearch also against Silva (v138).

### 2.4. Bacterial Diversity and Functions Predictions

In the multiple comparison bar chart, the feature occurrence was set to a minimum of 50%, Duncan’s Multiple Range Test (DMRT) was implemented, a log10 transformation was applied, and data standardization was performed with a p-value threshold of 0.05. Both alpha and beta diversity analysis were conducted using the R Vegan (2.6-8) package [41]. Boxplots with standard deviations display the Invsimpson index. Principal coordinate analysis (PCoA) was used to illustrate beta diversity and depicted the microbial communities’ taxonomic composition at the Phylum level. Plot visualizations were generated using the ggplot2 (3.5.1) package [42]. The functional metabolic potential of the microbial community was predicted using Functional Annotation of Prokaryotic Taxa (FAPROTAX) [43] and Phylogenetic Investigation of Communities by Reconstruction of Unobserved States 2 (PICRUSt2) [44] based on the full-length 16S rRNA gene sequencing data.

### 2.5. Generation of Random Forest Models

A random forest model depicted the link between the microbiota composition of the *S. canadensis* invasion community and varying warming temperatures by analyzing ASVs’ relative abundances against three temperature settings. Our dataset included 72 soil samples from this community, equally divided into training and testing groups (36 each). To ensure the robustness of our model, the number of trees (ntree) were all set to 1,000,000. Our analytical focus spanned multiple taxonomic levels, from Phylum to Genus, with specific attention given to the family level due to its demonstrated lower error rate (5.56%) in preliminary random forest classifications using the (importance = TRUE, proximity = TRUE) function in the R package randomForest [45].

Setting a random seed of 7 and achieving a low error rate of 2.78%, we proceeded with a random forest model at the family level. The model’s robustness was enhanced through a ten-fold cross-validation process using the rfcv() function in the randomForest package, eliminating less significant bacterial taxa and incorporating the most discriminant ones. This validation process helped us optimize the number of features, identifying 14 as the most effective for our model.

### 2.6. Construction of Bacterial Co-Occurrence Network

Network connections were determined by calculating Spearman’s rank correlations among ASVs. A correlation was considered valid if Spearman’s correlation coefficient (r) was both ≥0.6 and statistically significant at *p* < 0.01, indicating a strong and substantial association [46]. In our construction of the co-occurrence network, the Spearman rank correlations with a coefficient  >  |0.6| and a *p*-value < 0.01 were set. Based on the thresholds for within-module connectivity (Zi) and among-module connectivity (Pi), all nodes were categorized into four groups: peripherals (Zi  ≤  2.5 and Pi  ≤  0.62), connectors (Zi  ≤  2.5 and Pi  >  0.62), module hubs (Zi  >  2.5 and Pi  ≤  0.62), and network hubs (Zi  >  2.5 and Pi  >  0.62) [47]. The visualization and statistical analyses were conducted in R using the ggclusternet (0.1.0) [48] and igraph (2.1.1) packages [49].

## 3. Results

### 3.1. S. canadensis Invasion Community Responses to Warming

We collected 72 soil samples from the *S. canadensis* invasion community under three temperature conditions: ambient (T0), +1.15 °C (T1), and +1.86 °C (T2), to profile the bacterial community. Illumina sequencing yielded 5,676,811 high-quality sequences from 72 samples after amplification of the 16S rRNA gene’s V3-V4 region.

Soil bacteria showed significant variations in response to warming treatments across predominant phyla (Figure 1B). Acidobacteria, Actinobacteria, Aerophobetes, Euryarchaeota, Fibrobacteres, and Gemmatimonadetes significantly differed between the baseline (T0) and warmer conditions (T1 and T2). Armatimonadetes, Chloroflexi, and Elusimicrobia demonstrated a progressive change, significantly differing across all temperature steps (T0 to T1 to T2). In contrast, Chlorobi, Cyanobacteria, Firmicutes, and Hydrogenedentes show a pattern where T0 is different from T2, but T1 is not significantly different from T0. This suggests that the initial shift from T0 to T1 does not significantly alter these groups, but further change to T2 does. A group comprising Latescibacteria, Nitrospirae, Spirochaetae, and Thermotogae follows a unified trend. For these groups, T0 is statistically different from T2, indicating a notable response to these temperature extremes. A similarity between T0 and T1 or T1 and T2, wherever observed, indicates no statistically significant difference in the relative abundance of their response to these particular temperature treatments.

**Figure 2:**
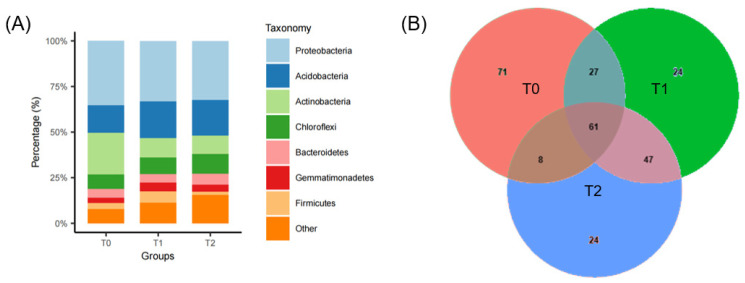
Microbial community composition and overlap across temperature treatments. (**A**) Composition of microbial communities across temperature treatments. Stacked bar chart illustrating proportional distribution of microbial taxa within each temperature group. (**B**) Venn diagram illustrating shared and unique microbial taxa present at each temperature condition, with size of each list denoted for each.

The bacterial community consisted of ASVs distributed among 41 phyla, 73 classes, 127 orders, 210 families, and 256 genera. At the phylum level, 92.10% of the ASVs fell within the top 10 phyla by relative abundance, namely Proteobacteria, Acidobacteria, Actinobacteria, Chloroflexi, Planctomycetes, Bacteroidetes, Verrucomicrobia, Gemmatimonadetes, Firmicutes, and Armatimonadetes. The abundances of bacterial phyla in various treatments are illustrated in Figure 2A. After screening with an average ASV abundance greater than 0.1%, there were 61 ASVs shared by all treatments. Two warming treatments introduced 48 ASVs to the soil bacteria of *S. canadensis* invasion community, of which 24 were recruited under each condition. In T1, the majority of these ASVs belonged to the phyla Acidobacteria (8), Proteobacteria (7), Firmicutes (5), Gemmatimonadetes (3), and Chloroflexi (1). In T2, the distribution was Proteobacteria (11), Bacteroidetes (4), Chloroflexi (4), Acidobacteria (2), Gemmatimonadetes (1), Nitrospirae (1), and Verrucomicrobia (1) (Figure 2B).

### 3.2. Bacterial Diversity and Predicted Functions Influenced by Warming

**Figure 3:**
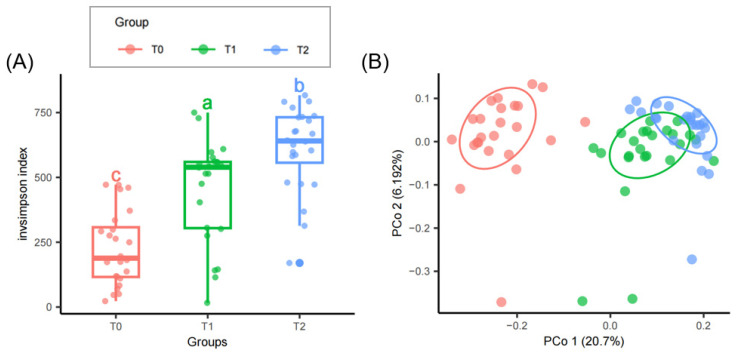
Microbial community diversity across temperature treatments. (**A**) Boxplot showing inverse Simpson index (invsimpson) variation for T0, T1, and T2, where higher values indicate lower species diversity. Statistically significant differences between treatments are indicated by different letters. (**B**) Principal Coordinates Analysis (PCoA) illustrating distinct microbial community clustering for each temperature treatment.

**Figure 4:**
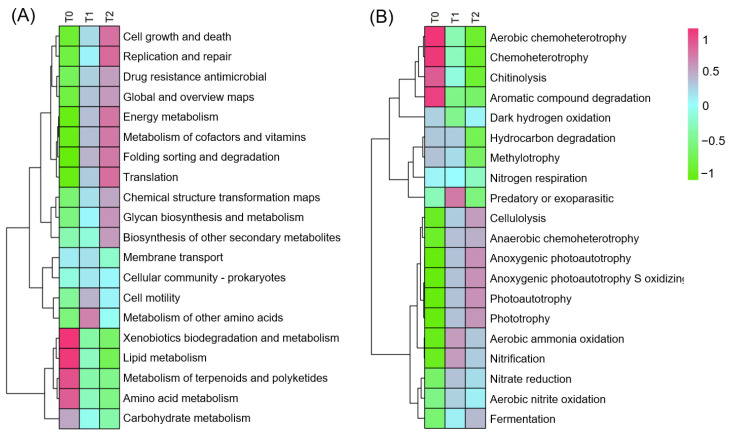
Comparative analysis of microbial metabolic functions and contributions to biogeochemical cycles across temperature treatments. (**A**) Heatmap illustrating PICRUSt2-inferred metabolic functions, showing differential potential within microbial communities across temperature treatments. (**B**) Heatmap visualizes FAPROTAX-predicted contributions of microbial taxa to Earth’s biogeochemical cycles under three temperature conditions, with color gradient from green to red reflecting normalized z-score.

The boxplot analysis (Figure 3A) reveals a gradient in microbial diversity across temperature treatments, with the lowest diversity at T0 and the highest at T2. Since the *y*-axis represents the inverse Simpson index, higher values indicate lower species diversity. Statistically, each temperature treatment community is significantly distinct from the others, confirming the impact of temperature on microbial composition in the invasion community. β-diversity shows that microbial communities adapt to temperature variations, leading to unique community structures (Figure 3B). A closer overlap between T1 and T2 suggests a gradual microbial composition shift with rising temperature, unlike the stark difference between T0 and T2. Metabolic functions, from cell growth and death metabolism to the biosynthesis of other secondary metabolisms, escalate with temperature (Figure 4A). These functions, which encompass fundamental cellular processes, antimicrobial resistance, and various biosynthetic pathways, are more active at higher temperatures (T2) compared to lower temperatures (T0), indicating up-regulation or increased prevalence of these processes at higher temperatures. Conversely, pathways involving the metabolism of xenobiotics, lipids, terpenoids, polyketides, amino acids, and carbohydrates decrease in activity. Functions predicted by Farprotax such as cellulolysis, anaerobic chemoheterotrophy, anoxygenic photoautotrophy, anoxygenic photoautotrophy (sulfur-oxidizing), photoautotrophy, and phototrophy tend to increase as temperature increases (Figure 4B). In contrast, processes like aerobic chemoheterotrophy, chemoheterotrophy, chitinolysis, and aromatic compound degradation are suggested to decrease with higher temperatures.

### 3.3. Predictive Model by Subsets of the Microbiota Biomarkers

**Figure 5:**
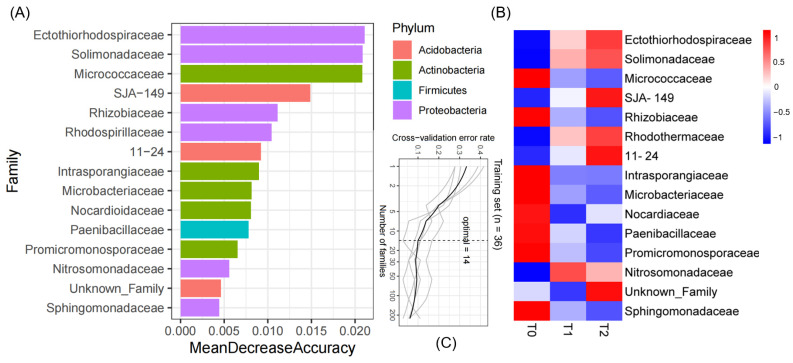
Machine learning analysis of soil microbial family importance under varying temperature treatments. (**A**) The bar chart represents the feature importance of bacterial families in the *S. canadensis* invasion community as determined by a random forest machine learning model, with the mean decrease in accuracy indicating the impact of each family on the model’s predictive performance, color-coded by phylum. (**B**) The heatmap displays the change in relative abundance of *S. canadensis* invasion community bacterial families across temperature treatments, with the color gradient reflecting the normalized z-score. (**C**) Performance curves of the random forest model during cross-validation.

Leveraging the random forest algorithm, our model correlates the *S. canadensis* invasion community microbial composition at the family level with temperature variations. From the model, 14 bacterial families were identified as the most discriminating features (Figure 5A), suggesting they significantly influence the community’s response to warming. These families might serve as indicators of how the *S. canadensis* invasion community microbial structure shifts with changes in temperature. The family Ectothiorhodospiraceae emerged as the top predictor, highlighting its abundance as highly temperature-sensitive and a potential biomarker for thermal impact analysis on microbial composition *S. canadensis* invasion community microbial composition (Figure 5B). Other families from the phyla Proteobacteria, Actinobacteria, and Acidobacteria also show varying degrees of importance, reflecting their roles in the community’s response to temperature.

At T0, a significant increase in relative abundance was observed in the following microbial families: Micrococcaceae, Rhizobiaceae, Intrasporangiaceae, Microbacteriaceae, Nocardiaceae, Paenibacillaceae, Promicromonosporaceae, and Sphingomonadaceae. At T1, the Nitrosomonadaceae family emerged as prominent biomarkers. In contrast, T2 exhibited a distinct set of microbial families, including Ectothiorhodospiraceae and Solimonadaceae, which showed elevated relative abundance.

### 3.4. Patterns of Interactions in Bacterial Co-Occurrence Network Under Warming

At T0, the network reaches peak complexity and diversity, with the highest number of bacterial taxa (692 nodes) and interactions (2559 edges) (Table 1). The average degree indicates that each bacterium at T0 interacts with an larger number of others, forming a dense network with closely knit clusters, as shown by the high mean clustering coefficient (0.34). Despite fewer clusters at T0 (*n* = 23), the abundance of interactions and the highest relative modularity (0.81) suggest these clusters are larger and more specialized, denoting a well-defined community structure. With rising temperatures to T1 and T2, network complexity noticeably declines. Evidenced by reduced bacterial taxa (539 and 340), interactions (853 and 399), and average degrees (3.17 and 2.35), this decline marks sparser connectivity. The clustering coefficient (0.30 and 0.29) remains relatively high, but with an increased number of clusters (60 and 72) and reduced modularity (0.43 and 0.27), the network appears to be more fragmented into smaller, less interconnected groups. These changes suggest a decline in the microbial community’s stability and robustness with rising temperatures (Supplementary Figure S1).

**Figure 6:**
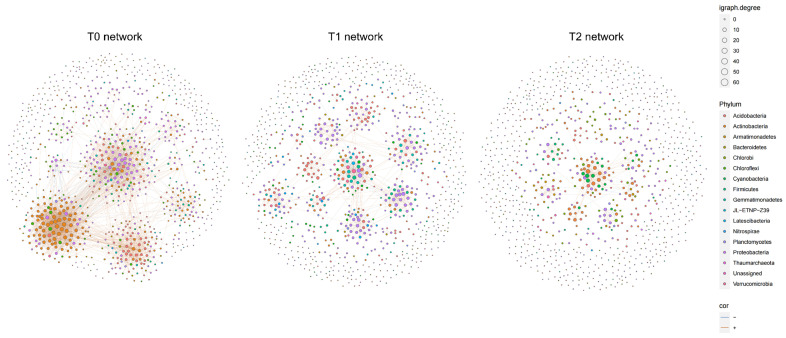
Co-occurrence network analysis of microbial communities under progressive thermal stress. Displayed are network graphs depicting the structure and interconnections of soil microbial communities in three temperature scenarios. Nodes within each network are differentiated by phylum and their connection degree is visualized by varying node sizes. “cor” denotes the correlation of edges, with positive and negative correlations indicated accordingly.

The T0 network’s microbial community shows rich bacterial diversity (Figure 6). Characterized by hub nodes, it spans phyla such as Proteobacteria (31.79%), Actinobacteria (26.01%), Acidobacteria (16.18%), Chloroflexi (8.38%), and Bacteroidetes (4.91%). As the temperature increases to T1, the network retains substantial diversity but undergoes reorganization. The T1 network transitions, revealing distinct sub-networks regulated by Proteobacteria (36.36%), Acidobacteria (23.75%), among others, marking a shift to a more modular structure. This suggests that despite sustained diversity, the shift to modularity possibly reflects adaptation to temperature changes, with key phyla central to interactions within sub-networks. At T2, the microbial community structure simplifies. Less diverse and complex, it has Actinobacteria (18.53%), Acidobacteria (15.88%), and Chloroflexi (10%) as predominant hubs.

**Figure 7:**
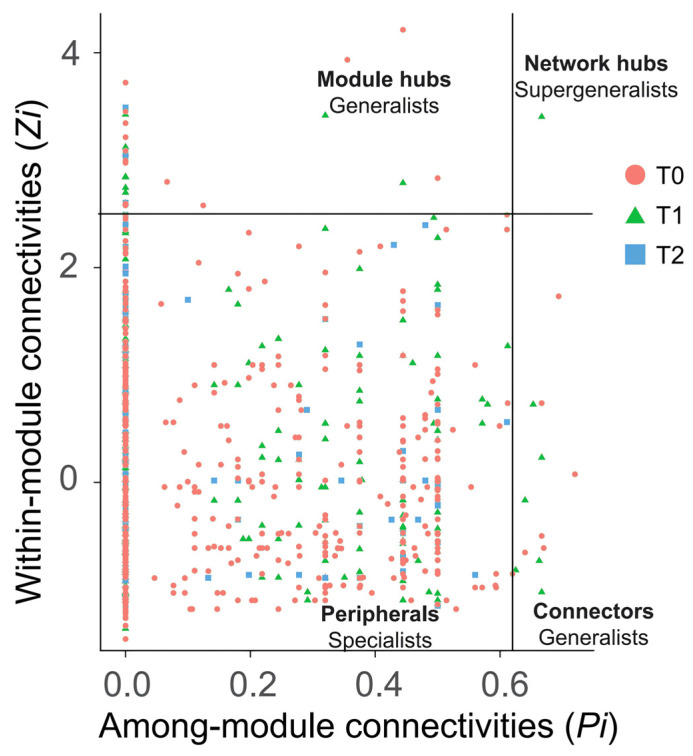
Zi-Pi plot showing the distribution of ASVs based on their topological roles. The scatter plot categorizes taxa based on their within-module connectivity (Zi) and among-module connectivity (Pi). Threshold values of Zi and Pi for categorizing OTUs were 2.5 and 0.62, respectively. Taxa are positioned as peripherals (specialists with few connections), module hubs (generalists within specific modules), connectors (generalists linking modules), or network hubs (super generalists central to overall network connectivity).

Connections of ASVs (nodes) within (Zi) and among (Pi) modules were determined in order to detect keystone microorganisms in the networks. Using threshold values of 2.5 (Zi) and 0.62 (Pi) [47], ASVs were categorized as peripherals, connectors, module hubs, or network hubs (Figure 7). In the T1 and T2 networks, a large percentage of ASVs (81.2% and 88.5%, respectively) lacked connections to any nodes outside of their respective modules (Pi = 0), suggesting that they were mostly peripheral nodes. T1 recruited more generalists than T2. These individuals in the T1 network serve as connectors (*n* = 6) and module hubs (*n* = 2), linking nodes within and between modules. There was only one node in the T1 network designated as a hub (a supergeneralist, ASV1160; Genus: Candidatus Lariskella), in contrast to the T2 network, where no node performed this role.

## 4. Discussion

This work presents a thorough examination of the *S. canadensis* invasion community microbial composition under three temperature circumstances. It uncovers the substantial influence of temperature fluctuations on both the diversity and function of the invasion community microbial population. The current study observed that elevating the temperature results in significant alterations in the composition and activity of bacterial populations. This finding aligns with the results of Xiong et al. [50], who reported similar disturbances in soil microbial dynamics due to short-term warming, underscoring temperature as a key environmental factor changing the structure of soil bacterial community.

Rising temperatures decreased bacterial community diversity, particularly affecting groups like Armatimonadetes and Chloroflexi. Microbial response differences were noted not only between baseline and warmed conditions but also between varying degrees of warming, indicating diverse adaptation patterns. Other groups, including Latescibacteria, also showed notable responses to temperature changes. This utilization of different microbes in the *S. canadensis* invasion community to mitigate different degrees of warming can be proven by the RF models showing that biomarkers predicted under different temperature treatments had distinct colonizing microbes (Figure 5B). Our initial hypothesis posited that warming would significantly alter the microbial community within the *S. canadensis* invasion community, leading to an increase in diversity and abundance of specific microbes that could enhance the plant’s invasiveness. Contrary to these expectations, our findings reveal that warming induces a decrease in microbial variety and interaction complexity could impede *S. canadensis*’s development and biomass buildup in the invasion community by preventing it from accessing these nutrients. These findings align with the previous phenotypic data, which indicates that warming, compared to the ambient treatment (T0), has a constraining effect on the diameter, height, biomass, and relative competitiveness of *S. canadensis* [13]. Notably, Cui’s results show that warming inhibits the growth of both native *A. argyi* and invasive *S. canadensis*, affecting key metrics such as plant height, root length, diameter, total biomass, root-to-shoot ratio, and specific leaf area [51]. Additionally, the decreased microbial diversity and interactions may not only hinder *S. canadensis* but could also negatively impact native *A. argyi* plants, leading to reduced nutrient availability and increased vulnerability to stressors [52].

Warming conditions lead to decreases in Acidobacteria, Firmicutes, and Gemmatimonadetes, potentially indicating that the element cycle and ecological function [53] have declined, impairing plant resistance to environmental stressors [54] and organic matter decomposition [55]. Despite seeming disadvantages, we speculate that *S. canadensis* in the invasion community might still exploit its associations with resilient microbes like the Ectothiorhodospiraceae, as revealed by biomarker analysis from RF models. These microbes, a family of purple sulfur bacteria, are pivotal in the sulfur cycle due to their unique photosynthetic ability in anaerobic conditions. Their ability to thrive in extreme conditions, such as temperature, salt concentration, and pH conditions [56], suggests a key role in stabilizing microbial communities in stressed soils.

Elevated temperatures lead to significant changes in the complexity and diversity of bacterial co-occurrence networks, resulting in a shift towards more specialized microbial communities and a reduction in generalist nodes. At T0, the microbial ecosystem was stable and complex. Rising temperatures to T2 simplified networks, reducing diversity and interaction, suggesting an adaptive recalibration for enhanced survival. Compared to the T2 network, T0 exhibited more complex interactions, a greater variety of bacterial phyla, and larger ASV modules with tighter connectivity and organization. Highly connected ASVs benefit the soil community, independent of their specific functions [57]. Simulated warming destabilizes microbial networks, particularly affecting temperature-sensitive groups like Acidobacteria and Actinobacteria [58].

Peripheral nodes dominate T1 and T2 networks, indicating a specialized group with limited external connectivity. This structure shows a microbial ecology adapted to specific conditions yet vulnerable to warming. The invasion community’s microbial adaptation to maintain network integrity and functionality during early temperature increases is seen from the number of 12 generalists in T0, which decreased to 8 connected within and among modules in T1. As temperatures rise (T2), the absence of super-generalists and network simplification indicate declining community resilience, potentially jeopardizing the health of both invasive and native plant and invasion success of *S. canadensis*. Previous studies have shown that generalist and specialist microbes impact the dynamics of microbial community structures differently [59]. A higher number of generalists in networks promotes balance, facilitating energy, information, and material exchanges among species [60]. Generalists exhibit broad environmental tolerances, while specialists have a narrower range of habitats and specific environment fitness [61]. Therefore, the decrease in the quantity of generalists and super-generalists post-warming is a predominant factor leading to the invasion obstacles of *S. canadensis*. In parallel, these changes in microbial networks may also adversely affect native plants *A. argyi*. Reduced microbial diversity and connectivity can limit nutrient availability and disrupt beneficial interactions that support native plant growth. This could lead to diminished competitive abilities for *A. argyi*, making it more susceptible to the pressures of invasion and environmental stressors. The impact of warming on T1 and T2 microbial networks reveals a dual facet: this duality highlights the fragile equilibrium in the *S. canadensis* invasion community and potential irreversible temperature tipping points, leading to drastic changes that could threaten both invasive and native plant species.

One limitation of this study is the age of the data, which were collected in June 2017. Given the rapid and ongoing changes in global climate, it is possible that microbial community responses to invasive species and warming conditions observed at that time may differ from those observed under current climate conditions. Future research using more recent data would be beneficial to validate these findings under current environmental conditions and assess any shifts that may have occurred in the intervening years.

## 5. Conclusions

In conclusion, this study underscores the profound impact of temperature fluctuations on the microbial ecosystem associated with the *S. canadensis* invasion community. Significant changes in microbial composition and activity were observed across varying levels of warming, highlighting a dynamic response that potentially hinders the invasiveness of *S. canadensis*. Particularly, the reduction in microbial diversity and the simplification of co-occurrence networks with rising temperatures suggest an ecosystem at a critical juncture, facing diminished resilience and increased vulnerability to warming stress. Our findings diverge from initial hypotheses, revealing a complex interplay between microbial community structure and plant invasion success, further complicated by the differential responses of generalist and specialist microbes to temperature changes. The emergence of specialized microbial groups under warming conditions, alongside the decline of generalist and super-generalist nodes, delineates a fragile equilibrium within the invasion community. This precarious balance not only affects *S. canadensis* but also threatens native plants like *A. argyi*, as reduced microbial support may limit their growth and competitive abilities. Given these insights, future research should focus on elucidating specific microbial functions and their direct implications for both *S. canadensis* and native species, aiming to develop targeted strategies for managing invasion risks and preserving native plant health in the context of global warming.

## Figures and Tables

**Table 1:** Topological properties of bacterial community in different temperature treatments.

Temperature Treatments	Temperature (°C)	Nodes	Edges	Average Degree	Mean Clustering Coefficient	Clusters	Relative Modularity
T0	29.47 ± 0.38	692	2559	7.40	0.34	23	0.81
T1	30.62 ± 0.36	539	853	3.17	0.30	60	0.43
T2	31.33 ± 0.37	340	399	2.35	0.29	72	0.27

## Data Availability

The original contributions presented in the study are included in the article/Supplementary Materials, further inquiries can be directed to the corresponding authors.

## Supplementary Materials

The following supporting information can be downloaded at: https://www.mdpi.com/article/10.3390/microorganisms12122415/s1. Figure S1: Co-occurrence network robustness of microbial communities under progressive thermal stress. File S1: Explanation of constructing invasion communities. References [13,62] are cited in the Supplementary Materials.
