# Supplementary material for: Effects of Increasing Temperature on Bacterial Community Diversity in Mixed Stands of Artemisia argyi and Solidago canadensis in Eastern China
Source: Microorganisms. 2024 Nov 25;12(12):2415. doi: 10.3390/microorganisms12122415 (PMC11677931; doi:10.3390/microorganisms12122415)
Supplement: Supplementary file 1 [file microorganisms-12-02415-s001.zip › File S1 explanation of constructing invasion communities.pdf]

S1.1. Constructing Invasion Communities by Stages: Gradual Simulation from Early Presence to Dominance

Plant invasion is, in essence, a continuous process, with varying degrees of invasion at a single site often influenced simultaneously by environmental changes [1]. Therefore, to better understand the relationship between environmental changes and plant invasion, it is crucial to examine how environmental shifts impact the invasion process across different invasion intensities, rather than focusing solely on specific invasion stages [2]. Our methodology revolves around "Mimicking Real-World Invasion Community through Integrating Multiple Invasion Stages" to realistically simulate invasion community under varying temperatures. Six stages represent the invasion progression, from an early single-species stage to a dominance stage. Early Stage (S1A3): Minimal invasion, with one S. canadensis seedling and three A. argyi seedlings. Below Intermediate Stage (S2A3): A slight increase in S. canadensis, with two seedlings alongside three A. argyi seedlings. Intermediate Stage (S3A3): A balance between invasive and native species, with three seedlings of each. Above Intermediate Stage (S3A2): S. canadensis begins to dominate, with three of its seedlings and two A. argyi seedlings. Dominant Stage (S3A1): S. canadensis clearly dominates, with three of its seedlings and one A. argyi seedling. Balanced Invasion Stage (S2A2): Equal representation, with two seedlings of each species.

1. Theoharides, K.A.; Dukes, J.S. Plant Invasion across Space and Time: Factors Affecting Nonindigenous Species Success during Four Stages of Invasion. *New Phytol.* **2007**, *176*, 256–273, doi:10.1111/j.1469-8137.2007.02207.x.
2. Ren, G.; Yang, B.; Cui, M.; Dai, Z.; Xiang, Y.; Zhang, H.; Li, G.; Li, J.; Javed, Q.; Du, D. Warming and Elevated Nitrogen Deposition Accelerate the Invasion Process of Solidago Canadensis L. *Ecological Processes* **2022**, *11*, 62, doi:10.1186/s13717-022-00407-8.
