# Supplementary figures and images for: Effects of Increasing Temperature on Bacterial Community Diversity in Mixed Stands of Artemisia argyi and Solidago canadensis in Eastern China
Source: Microorganisms. 2024 Nov 25;12(12):2415. doi: 10.3390/microorganisms12122415 (PMC11677931; doi:10.3390/microorganisms12122415)

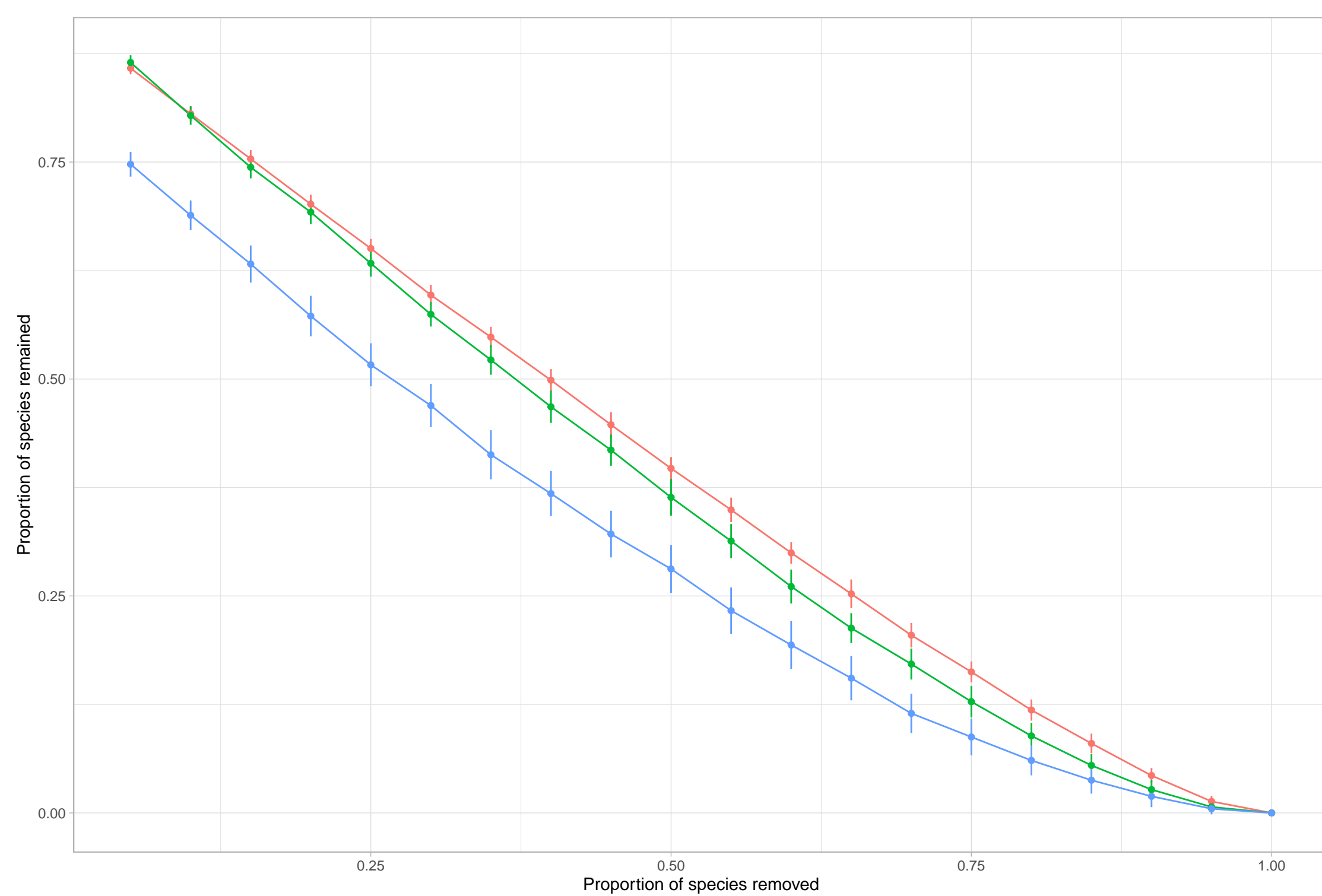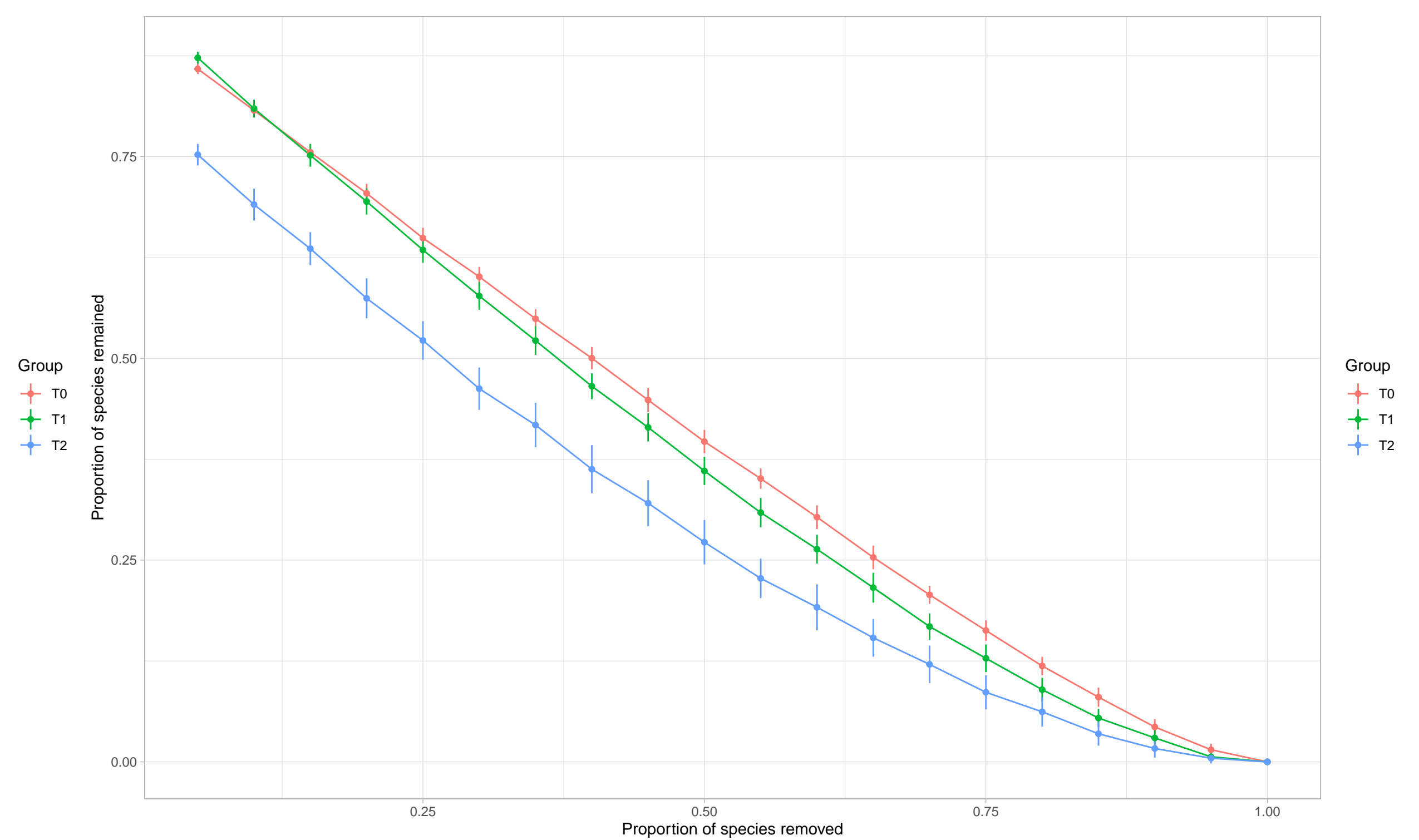

Supplement: Supplementary file 1 [file microorganisms-12-02415-s001.zip › Figure S1 Network Robustness.pdf]
